# Regulation Law of Tempering Cooling Rate on Toughness of Medium-Carbon Medium-Alloy Steel

**DOI:** 10.3390/ma17010205

**Published:** 2023-12-30

**Authors:** Chao Yang, Tingting Xu, Hongshan Zhao, Chundong Hu, Han Dong

**Affiliations:** 1 School of Materials Science and Engineering, Shanghai University, Shanghai 200444, China; alphayangchao@shu.edu.cn (C.Y.); boyushankf@126.com (H.Z.); donghan@shu.edu.cn (H.D.); 2 Zhongyuan Special Steel Co., Ltd., Jiyuan 459000, China; xuting_1@163.com; 3 Zhejiang Institute of Advanced Materials, Shanghai University, Jiaxing 314100, China

**Keywords:** temper embrittlement, medium-carbon medium-alloy steel, martensite, bainite, toughness

## Abstract

Temper embrittlement is a major challenge encountered during the heat treatment of high-performance steels for large forgings. This study investigates the microstructural evolution and mechanical properties of Cr-Ni-Mo-V thick-walled steel, designed for large forgings with a tensile strength of 1500 MPa, under different tempering cooling rates. Optical microscopy (OM), scanning electron microscopy (SEM), and electron backscatter diffraction (EBSD) were employed to analyze the microstructural features. The results demonstrate that the embrittlement occurring during air cooling after tempering is attributed to the concentration of impurities near Fe_3_C at the grain boundaries. The low-temperature impact toughness at −40 °C after water quenching reaches 29 J due to the accelerated cooling rate during tempering, which slows down the diffusion of impurity elements towards the grain boundaries, resulting in a reduced concentration and dislocation density and an increased stability of the grain boundaries, thereby enhancing toughness. The bainite content decreases and the interface between martensite and bainite undergoes changes after water quenching during tempering. These alterations influence the crack propagation direction within the two-phase microstructure, further modifying the toughness. These findings contribute to the understanding of temper embrittlement and provide valuable guidance for optimizing heat treatment processes to enhance the performance of high-performance steels in large forgings.

## 1. Introduction

Steels for large forgings undergo temper embrittlement during the heat treatment process at temperatures ranging from 250 °C to 400 °C and 500 °C to 650 °C. Temper embrittlement typically manifests in two types: reversible and irreversible. Research on both types of temper embrittlement has primarily focused on the segregation of harmful elements such as phosphorus, sulfur, tin, antimony, and arsenic at the grain boundaries during tempering or aging processes. This segregation reduces the bonding strength of iron atoms at the grain boundaries, making cracks prone to initiate and propagate along the grain boundaries, ultimately leading to intergranular fracture [1]. The sensitivity of elements in steel to temper embrittlement is in the order: P > Sn > Sb ≈ As. Additionally, alloying elements such as Ni, Cr, and Mn are significant contributors to temper embrittlement. In the absence of these elements, temper embrittlement is generally not induced, and the order of alloying elements in terms of embrittlement capability is Mn ≈ Si > Cr > Ni. The impact of a single alloying element on temper embrittlement in steel is not significant, but the combined addition of alloying elements greatly promotes the occurrence of temper embrittlement. Attempts have been made to synthesize the effects of alloying elements influencing temper embrittlement into a single coefficient to assess the sensitivity of steel to temper embrittlement. In the 1970s, Bruscato [2] introduced a brittleness factor, X = (10P + 5Sb + 4Sn + As) × 102, which can be used to predict the brittleness sensitivity of metals. Watanabe [3], in conjunction with experiments on plates and forgings, identified a brittleness sensitivity factor, J = (Si + Mn)(P + Sn) × 104. As X and J values increase, steel transitions towards embrittlement. Bandyopadhyay [4] studied the precipitation behavior of carbides during the tempering process of Ni-Cr-V and Ni-Cr-Mo-V steels. They explained that molybdenum enhances the cohesion of grain boundaries, while phosphorus is less prone to segregate to grain boundaries. Begley [5] investigated the temper embrittlement sensitivity and crack propagation rate characteristics of Ni-Cr-Mo-V steel, noting a sharp increase in fracture toughness at room temperature. Sang-Gyu Park [6] employed thermodynamic calculations to assess the influence of Cr, Mn, and Ni on temper embrittlement in low-alloy steel for nuclear power applications from the perspectives of P diffusion rate and C activity. To suppress reversible temper embrittlement, alloying with Mo and W has long been considered the most effective method. Briant [7] described two primary mechanisms of temper embrittlement: delayed effects during P segregation and increased boundary cohesion strength with the addition of Mo. Petrov and Tsukanov [8] associated irreversible temper embrittlement with the precipitation of carbide elements at grain boundaries, proposing that alloying with Mo and other carbide-forming elements (e.g., Cr) shifts the temperature range of irreversible temper embrittlement from 250 °C to 400 °C. Yang [9] investigated M152 martensitic heat-resistant steel with slow quenching and found that the continuous distribution of M_23_C_6_ along the original austenite grain boundaries and M_2_C along the residual austenite film is the cause of a sharp decrease in toughness. Lei [10] determined, through the measurement of internal friction temperature curves, that high-temperature temper embrittlement belongs to the category of α-phase aging and exhibits reversibility. Kim [11] studied the effects of vanadium and carbides on the temper embrittlement of Cr-Mo steel.

This paper investigates the embrittlement phenomenon occurring during the high-temperature tempering of a newly developed medium-carbon, medium-alloy Cr-Ni-Mo-V steel by our research team. This study employs optical microscopy and scanning electron microscopy to observe the structural characteristics and utilizes electron backscatter diffraction for quantitative analysis of grain boundary orientation differences; the impact of microstructure on the temper embrittlement phenomenon in large forgings of Cr-Ni-Mo-V steel was evaluated from the perspective of grain boundary characteristics. Additionally, the phenomenon of reduced susceptibility to temper embrittlement due to the increase in the volume fraction of martensite was analyzed by altering the tempering cooling rate, and we explored the fundamental mechanisms of how the tempering cooling rate affects the organizational state for enhancing impact toughness.

## 2. Experimental Materials and Methods

**Figure 1:**
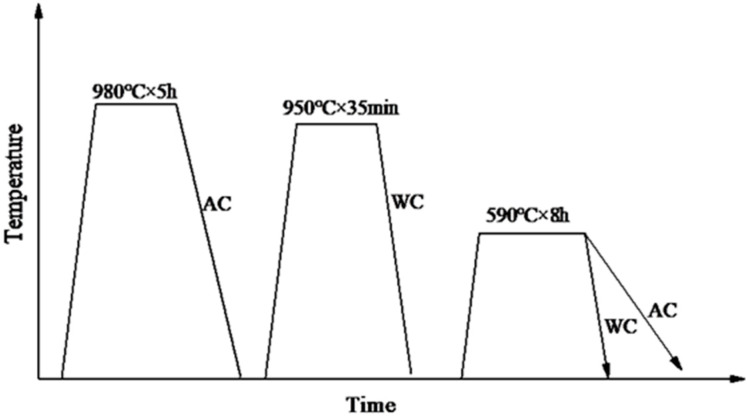
The diagrammatic sketch of heat treatment process.

**Figure 2:**
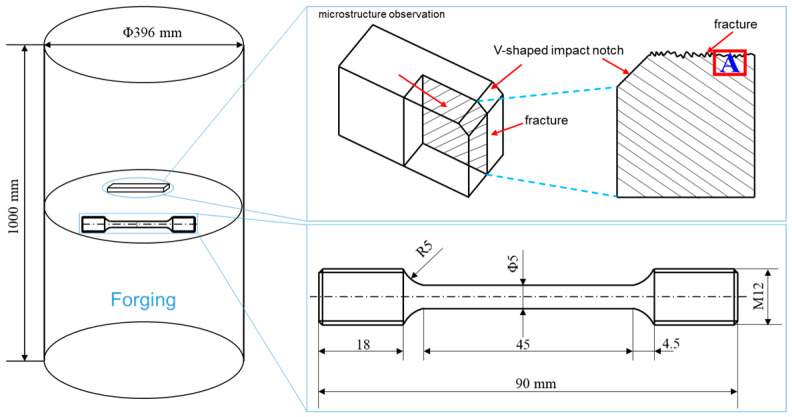
Forging size and sampling position and size of tensile specimen and impact specimen.

The experimental material was obtained from the cross-section of a Φ396 mm Cr-Ni-Mo-V steel. The specimens were initially in the as-forged and annealed state. The compositions of the sample steel used in this study are listed in Table 1. The cross-sectional samples were subjected to quenching and tempering processes, as illustrated in Figure 1. For both quenching and tempering heat treatment processes, the samples were placed in the furnace once the desired temperature was reached, with a heating rate of 10 °C/min. After tempering, specimens were sampled at half the radius of the cross-section for both air cooling (TAC) and water cooling (TWC) conditions, and these samples were mechanically processed to determine the strength and Charpy impact toughness at −40 °C. V-notch impact specimens with dimensions of 10 mm × 10 mm × 55 mm were utilized for this purpose. During the tensile testing, strain was measured using an extensometer with a gauge length of 25 mm, and the strain rate was 1 mm/min. The size of the tensile specimen in the experiment is shown in Figure 2. Samples with dimensions of 20 mm × 20 mm × 20 mm were mechanically polished, corroded in a 4% nitric acid ethanol solution, and observed using an optical microscope (Carl Zeiss Axio Imager.M2m, CarlZeiss, Jena, Germany) produced by Leica. The microstructure and fracture analysis of the specimens under both conditions were collected using a Apreo 2S HiVac scanning electron microscope (SEM) produced by FEI (Thermo Fisher Scientific, Waltham, MA, USA). To investigate the relationship between crack initiation, crack propagation, and microstructure, the fracture surfaces of the −40 °C Charpy impact specimens were studied below the area of the fracture surface, as shown in Figure 2, and subjected to electron backscatter diffraction (EBSD) analysis. Samples for EBSD studies were vibratory polished using 0.02 μm colloidal silica polishing solution. EBSD data were obtained using the Oxford EBSD system equipped with Channel 5 software (Oxford-HKL) and AZtecCrystal v2.1 for post-processing-oriented data analysis to assess the differences in the segregation behavior of tempered martensite under different tempering cooling rates. For the observation of carbide precipitation at grain boundaries under the two tempering cooling conditions, a JEM-2010F (JEOL, Tokyo, Japan) transmission electron microscope (TEM) was employed.

## 3. Results and Discussion

### 3.1. Mechanical Properties and Microstructure of Tempering Cooling Methods

The mechanical properties of the cross-sectional samples of the experimental steel after heat treatment are presented in Table 2. The air-cooled specimen exhibited a yield strength and tensile strength of 1145 MPa and 1422 MPa, respectively. For the water-cooled and tempered specimen, the yield strength and tensile strength were 1245 MPa and 1480 MPa, respectively. The strengths under both conditions are comparable. However, the impact energy of the water-cooled and tempered specimen is significantly higher than that of the air-cooled specimen, confirming the reversibility of temper embrittlement in this steel. The Charpy impact energy for the air-cooled specimen at −40 °C is 19 J, lower than the 29 J observed for the water-cooled and tempered state, indicating a substantial change in toughness. To understand the factors influencing toughness, it is essential to consider the pattern of crack propagation within the crystal lattice.

**Figure 3:**
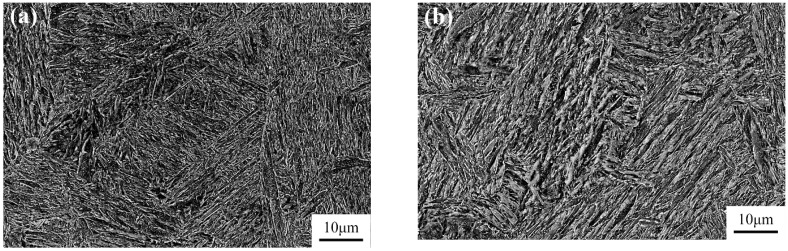
Two cooling methods’ SEM microstructure: (**a**) air cooling and (**b**) water cooling.

**Figure 4:**
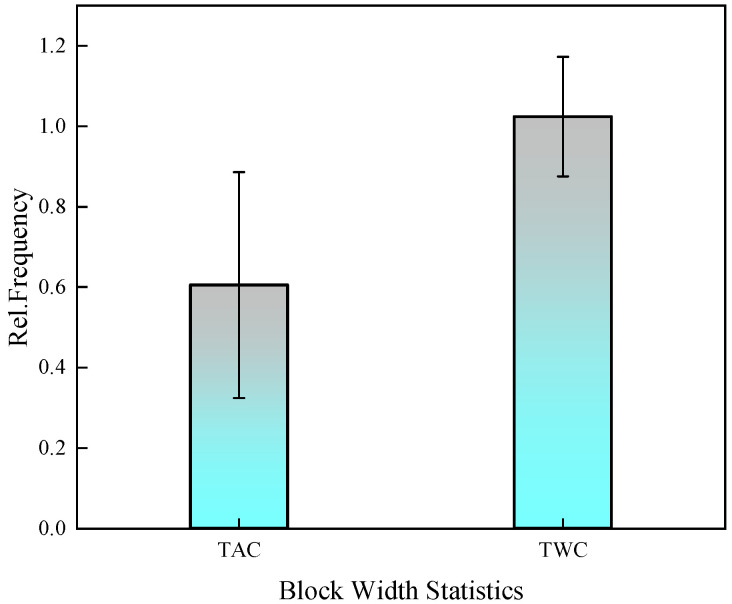
Comparison of block widths in air-cooled and water-cooled martensite.

Figure 3 depicts the microstructure of the experimental steel after tempering at 590 °C, followed by air cooling and water cooling. Figure 3a displays the SEM morphology for air cooling, while Figure 3b presents the SEM morphology for water cooling. Upon observing the microstructure, it is evident that both air-cooled and water-cooled structures consist of a mixture of tempered martensite and bainite. In the air-cooled condition, the martensite laths mainly appear as long needle-shaped structures within the microstructure, with larger grain size. Scanning electron microscopy reveals that the carbides precipitated after air cooling are primarily located at the grain boundaries. In contrast, the water-cooled microstructure is more uniform, with martensite laths arranged in a plate-like manner. Fine carbides are observed to be dispersed within the lath matrix, as shown in Figure 4. The average block width of martensite is 0.6 μm for air cooling (TAC) and 1 μm for water cooling (TWC), as measured under SEM.

**Figure 5:**
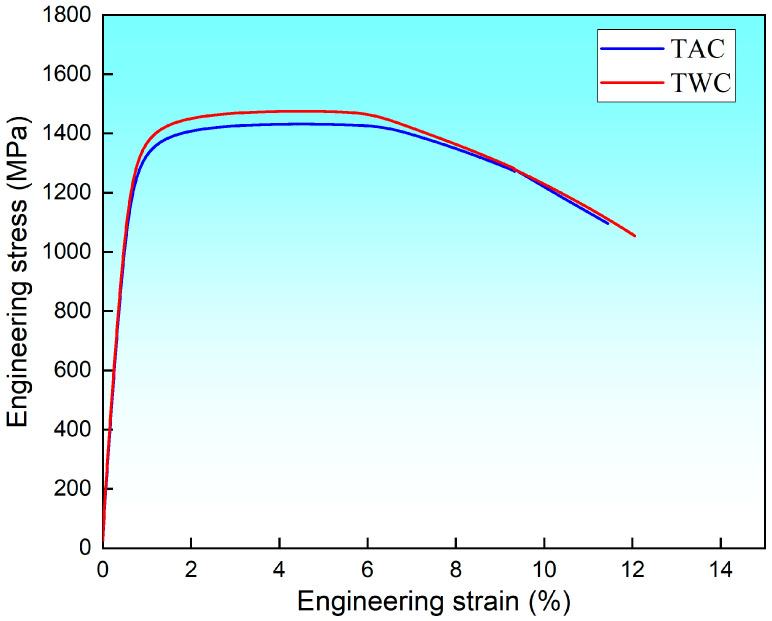
Stress–strain curves under two different cooling methods.

**Figure 6:**
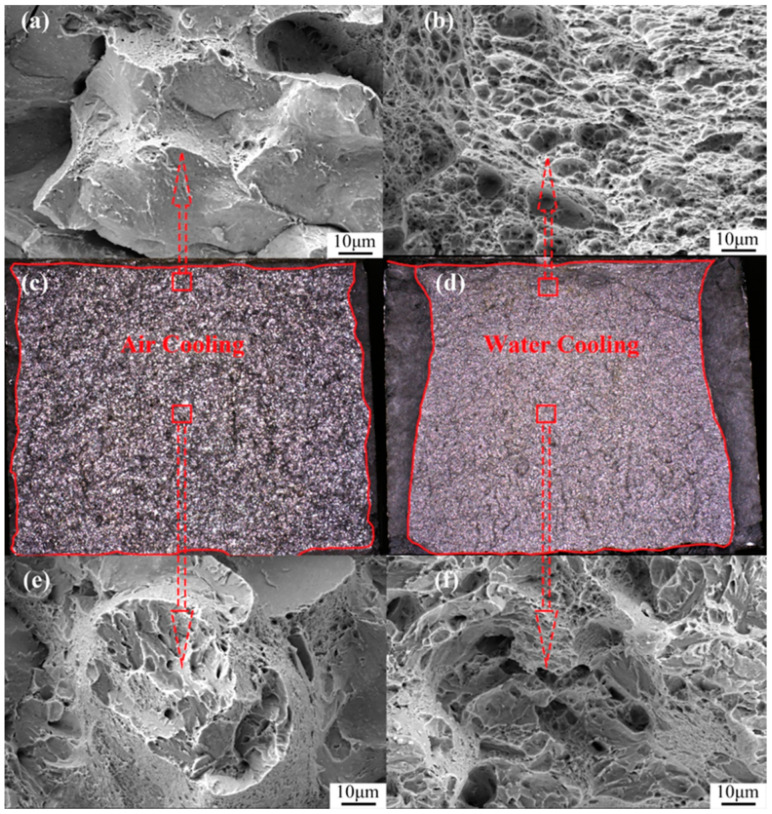
Different tempering cooling methods’ −40 °C low-temperature impact fracture morphology: (**a**) air-cooled fracture initiation zone SEM, (**b**) water-cooled fracture initiation zone SEM, (**c**) air-cooled macrofracture zone, (**d**) water-cooled macrofracture zone, (**e**) air-cooled fracture extension zone SEM, and (**f**) water-cooled fracture extension zone SEM.

Figure 5 shows the stress–strain curves after tensile testing under two different cooling methods. It can be observed that after water cooling, there is a slight increase in yield strength, tensile strength, and elongation. Specifically, the tensile strength increased by about 58 MPa, and the yield strength increased by approximately 100 MPa. Figure 6 illustrates the macroscopic and SEM morphology of the fracture surfaces at −40 °C after tempering and subsequent air cooling and water cooling of the experimental steel. By comparing the macroscopic morphology, it is evident that the air-cooled fracture surface (Figure 6a) appears bright white, with a relatively narrow shear lip. Under SEM, the fracture initiation zone (Figure 6c) exhibits intergranular fracture, and in the fibrous region (Figure 6e), both intergranular and cleavage fractures are observed. The impact toughness is significantly low, measuring 19 J.

**Figure 7:**
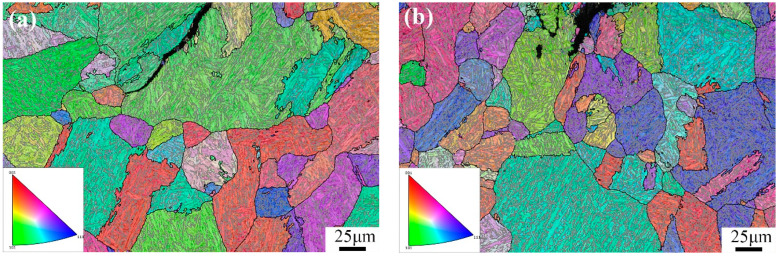
EBSD micrograph of protoaustenite grain reconstruction for (**a**) tempering air cooling and (**b**) tempering water cooling.

In contrast, the macroscopic morphology of the water-cooled fracture surface (Figure 6b) shows a typical wider shear lip. Moreover, under SEM, the fracture initiation zone (Figure 6d) displays ductile dimples, and in the fibrous region (Figure 6f), ductile dimples and cleavage facets are observed, indicating quasi-cleavage. The toughness is improved by 10 J compared to air cooling, reaching 29 J. Figure 7 presents the EBSD results of the microstructure after tempering and subsequent air cooling and water cooling, reconstructed using the AZtecCrystal v2.1 software. It can be observed that after air cooling, the cracks propagate along the grain boundaries, while after water cooling, the cracks propagate transgranularly.

### 3.2. Impact of Cooling Rate on Crack Propagation

For large forgings, in order to reduce the thermal stress during the tempering process in the temperature range of 400 °C to 650 °C, a slow cooling method is generally employed, corresponding to the temper-embrittlement-sensitive zone of steel. The cooling rate significantly influences crack propagation. At slower cooling rates, cracks tend to propagate along grain boundaries, and the propagation of intergranular fractures is related to the weakening of interfaces. The preferred path for crack propagation is at defect locations or areas of interface weakening. Fine needle-shaped martensite can lead to unstable intergranular structures, reducing the energy required for crack absorption. In contrast, at faster cooling rates, martensite exists in a plate-like form, and the aspect ratio of the laths decreases, increasing the probability of crack transgranular propagation and improving toughness [12].

**Figure 8:**
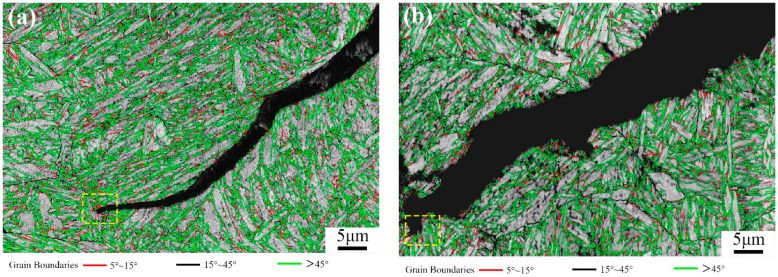
Band contrast (BC) maps depicting boundary distribution in (**a**) TAC and (**b**) TWC (red line: 15° > θ > 5°, black line: 45° > θ > 15°, green line: θ > 45°).

**Figure 9:**
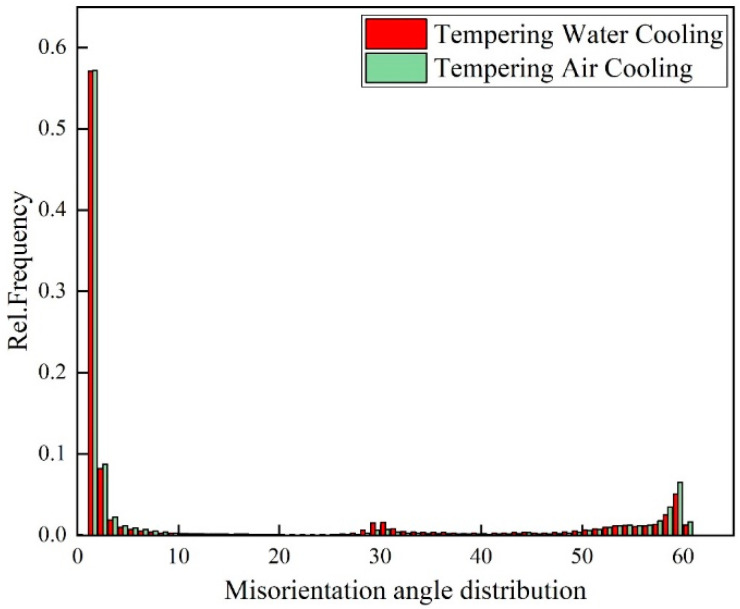
Relative frequency of grain boundary structure by different tempering cooling methods.

The AZtecCrystal software was utilized to analyze the distribution of grain boundaries near the cracks. Figure 7 shows the misorientation distribution of grain boundaries near the cracks under two conditions. Low-angle grain boundaries (LAGBs) with misorientation angles from 2° to 15° are represented by red lines, while high-angle grain boundaries (HAGBs) with misorientation angles from 15° to 45° are represented by black lines, indicating a minimal difference between air-cooled and water-cooled tempering conditions. Boundaries with angles greater than 45° are denoted by green lines. Figure 8 compares the relative frequencies of LAGBs and HAGBs under two different tempering cooling rates, revealing a similar frequency for both low- and high-angle grain boundaries. Previous studies have suggested that HAGBs hinder crack propagation [13,14,15]. Additionally, research has indicated that LAGBs play a crucial role in both strength and toughness. The high dislocation density generated in the overcooled austenite during processing can be inherited by martensite, forming sub-grains through dynamic recovery. Such low-angle grain boundaries have an inhibiting effect on crack propagation. In the yellow dashed box in Figure 8, low-angle grain boundaries are concentrated at the crack arrest position. The frequency of grain boundaries does not change significantly under different tempering cooling rates for the experimental steel. However, after water cooling and tempering, as the martensite lath bundles widen, the position of low-angle grain boundaries changes. Compared to the air-cooled state, more low-angle grain boundaries appear between the lath bundles rather than around the original austenite grain boundaries. Figure 9 shows the relative frequency of grain boundary structure by different tempering cooling methods; no significant changes were observed in the boundary frequency under the two different cooling methods.

**Figure 10:**
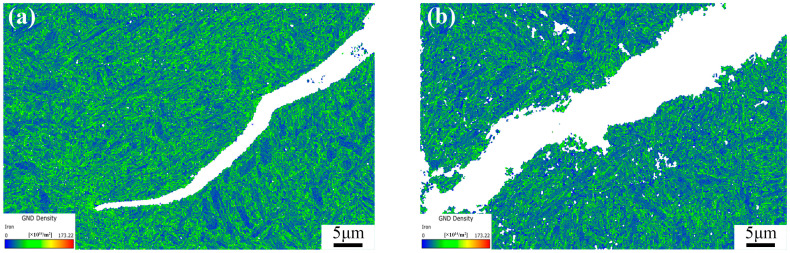
GND density micrographs for (**a**) TAC and (**b**) TWC.

**Figure 11:**
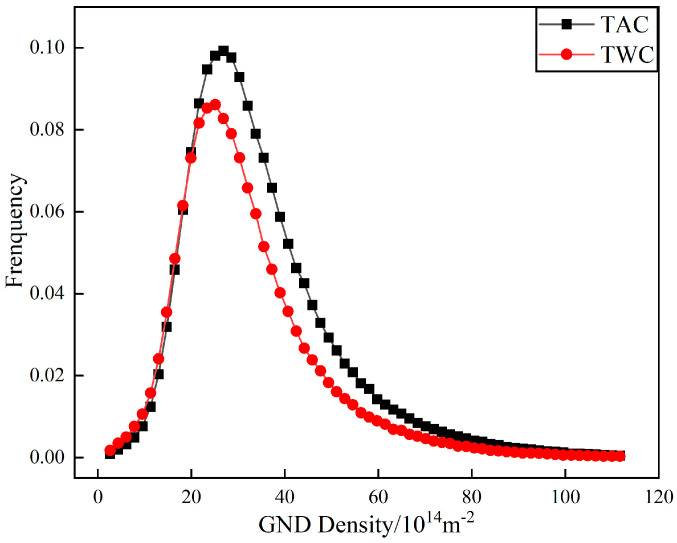
Geometrically necessary dislocation (GND) density distribution.

Geometrically necessary dislocations (GNDs) can provide quantitative information about localized plastic deformation. In some studies, this method can also be used to characterize the distribution of orientation deviations in cracks. Through the AZtecCrystal software, GND distributions under different tempering cooling rates are color-coded, as shown in Figure 9. Blue represents regions with lower dislocation orientation deviations, while green represents regions with high geometrically necessary dislocation densities. Larger GNDs are distributed near the crack, and at the crack tip in Figure 10a, there is a higher density of geometrically necessary dislocations. It can be observed that GNDs tend to be distributed at many low-angle grain boundaries, as low-angle grain boundaries hinder the slip of dislocations, leading to the accumulation of GNDs. In contrast, high-angle grain boundaries do not have a significant distribution of GNDs, indicating a strong ability to absorb dislocations. They are potential locations for crack initiation, and when cracks coincide with regions of high stress concentration and high-angle boundaries, crack initiation and propagation are accelerated [16]. Using the HKL Channel 5 software, GNDs were geometrically calculated for the two conditions, as shown in Figure 11. It can be seen that the frequency of GNDs is lower after tempering and water cooling, indicating more stable low-angle and low-energy grain boundaries. These boundaries resist sliding, thereby enhancing toughness.

**Figure 12:**
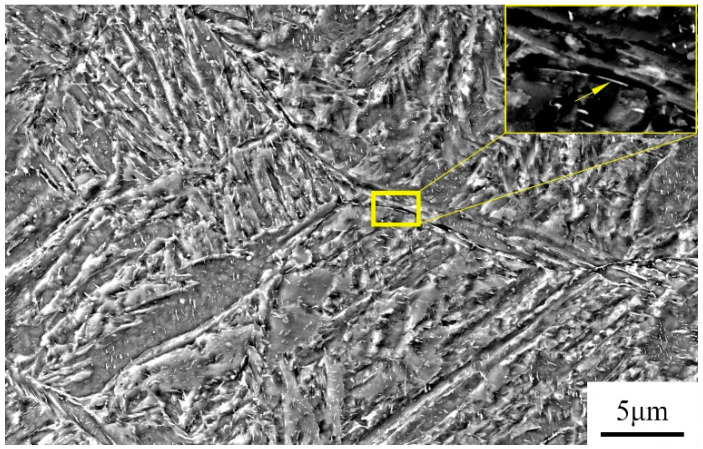
Microstructure with points of intergranular particle analyzed with EDS (see results in Table 2).

**Figure 13:**
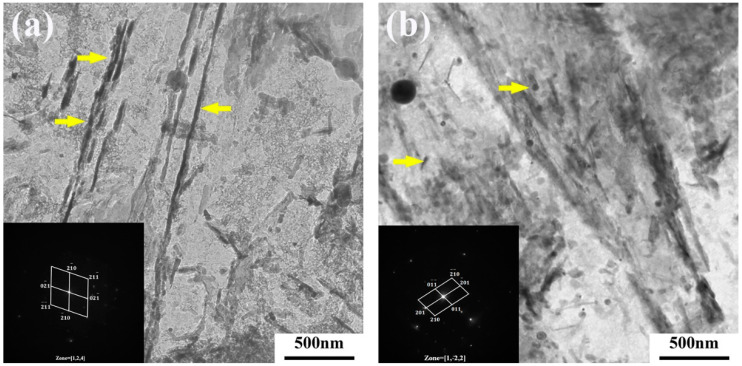
(**a**) Air-cooled and (**b**) water-cooled TEM characterization of Fe_3_C.

On the other hand, during the tempering process, the precipitation of Fe_3_C at grain boundaries and the segregation of impurity atoms lead to interface weakening and brittle fracture, as depicted in Figure 12. Fe_3_C exists in a rod-like form along the grain boundaries, and Table 2 provides the EDS analysis of Fe_3_C at the grain boundaries. The diffusion rate of impurity atoms at the grain boundaries is higher than that of iron atoms because there are more vacancies at the grain boundaries, which can promote dislocation migration. Consequently, there is a higher density of dislocations and impurity content at the grain boundaries, and the pinning effect of impurities can easily result in temper embrittlement [17,18]. McMahon proposed that during tempering in the embrittlement temperature range, Fe_3_C precipitates along the grain boundaries, and since impurities have low solubility in Fe_3_C, impurities are rejected and concentrated at the interface during carbide precipitation. This creates favorable pathways for crack propagation along the interface, constituting a non-equilibrium segregation process [19,20]. Rapid cooling after tempering suppresses the process of impurity element enrichment and segregation. The rod-like Fe_3_C transforms into a spherical shape, as shown in Figure 12. Table 3 shows the EDS of Fe_3_C in the yellow box in Figure 12. Figure 13a illustrates the rod-like Fe_3_C near the grain boundaries observed using TEM in the air-cooled state, while Figure 13b displays the spherical Fe_3_C near the grain boundaries observed using TEM in the water-cooled state. This process helps avoid the occurrence of the second type of temper embrittlement or eliminates already-formed temper embrittlement.

### 3.3. Influence of Cooling Rate on M/B Microstructure

**Figure 14:**
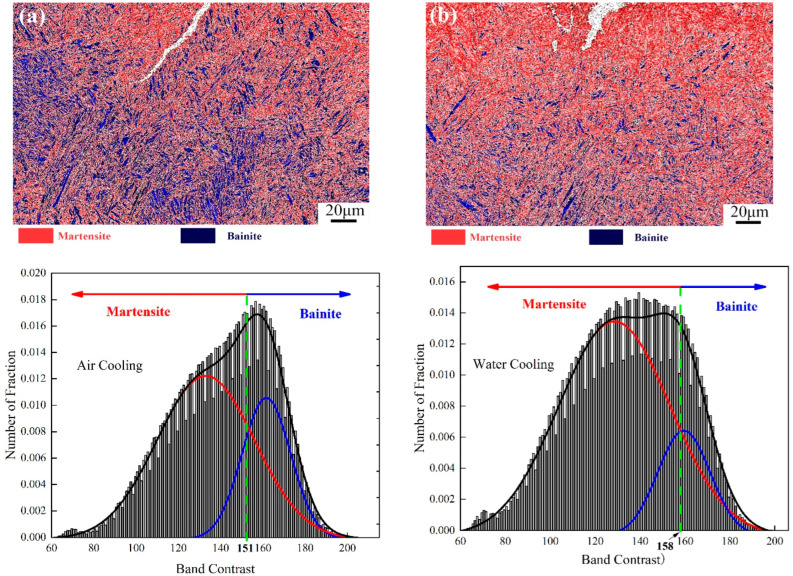
(**a**) Distribution of martensite and bainite complex structures in air-cooled and (**b**) water-cooled states.

The samples after two tempering cooling methods were characterized using EBSD, and quantitative analysis of the BCC phase was performed. Based on the BC values from the Kikuchi model and using Gaussian multi-peak fitting, the fraction distribution of martensite and bainite was analyzed [21,22,23]. The thresholds for differentiating martensite and bainite were set in the Gaussian plot by intersecting the Gaussian curves, as shown in Figure 14. Combining the BC plot to calculate the proportions of each phase, Table 4 indicates that in the microstructure of the air-cooled sample after tempering, martensite occupies approximately 60 vol%, and bainite occupies about 40 vol%. After tempering and water cooling, the microstructure comprises approximately 67% martensite and 33% bainite. Research by Edwards suggests that the martensite–bainite mixed structure in medium-carbon Cr-Ni-Mo-V steel exhibits good toughness. Additionally, when the martensite comprises ≤25% bainite on the martensite matrix, the toughness improves under the same strength conditions. This is because when cracks propagate through the two-phase structure, the different toughness of the phases causes a change in the direction of crack expansion. The optimal microstructure state, where bainite accounts for close to 25% after tempering and water cooling, leads to an improvement in toughness compared to the air-cooled tempering state. This is attributed to the fact that during rapid tempering and cooling, bainite forms before martensite, pre-dividing the grains and effectively refining the grain structure, resulting in improved toughness.

## 4. Conclusions

This study investigated temper embrittlement in medium-carbon alloy steel during the production of large forgings, focusing on both mechanical properties and microstructural aspects. The results can be summarized as follows:

1. After air-cooling tempering, the fracture surface is dominated by intergranular fracture, while after tempering and water cooling, quasi-cleavage fracture is predominant. The distribution of high- and low-angle grain boundaries shows minimal differences between the two states. However, tempering and water cooling result in lower dislocation density, and the improvement in toughness is related to the decrease in dislocation density.
2. The second type of temper embrittlement observed in medium-carbon alloy steel large forgings during high-temperature tempering at 590 °C is attributed to the concentration of impurities near the grain boundaries, especially around Fe_3_C. Tempering and water cooling suppress this segregation, thereby eliminating the already-occurring temper embrittlement. At the same time, with an increase in tempering rate, the morphology of Fe_3_C transforms from elongated rods to granular.
3. The impact toughness is higher after tempering and water cooling compared to air-cooling tempering. The microstructure in both states consists of a mixture of martensite and bainite. In the air-cooled state, martensite plates exist in the form of long needles, while in the water-cooled state, the structure consists of uniformly distributed martensite plates. The reduction in bainite content during tempering and water cooling approaches the optimal ratio for martensite–bainite mixed structures, contributing to the enhanced toughness.
4. For the reversible temper brittleness issue that occurs in large forgings, it can be mitigated by using water cooling, i.e., increasing the cooling rate during tempering, to enhance toughness. This method is applicable to large Cr-Ni-Mo-V forgings.

## Figures and Tables

**Table 1:** Chemical compositions of the sample steels used in this study (in wt.%).

C	Si	Mn	P	S	Ni	Cr	Mo	V	As	Sn	Pb	Sb	Bi
0.26	0.2	0.6	0.005	0.006	3.0	1.0	0.6	0.2	<0.008	<0.006	<0.005	<0.006	<0.005

**Table 2:** Mechanical properties after different tempering cooling methods.

Heat Treatment Process	*R*_p0.1_/MPa	*R*_m_/MPa	*A*(%)	*Z*(%)	*KV*_2_@−40 °C (J)
980 °C × 5 h, AC + 950 °C × 35 min, WC + 590 °C × 8 h, AC	1145 ± 23	1422 ± 28	11 ± 0.4	53 ± 1.5	19 ± 3
980 °C × 5 h, AC + 950 °C × 35 min, WC + 590 °C × 8 h, WC	1245 ± 8	1480 ± 10	12 ± 1.2	54 ± 0.6	29 ± 5

**Table 3:** EDS analysis results of position in Figure 9.

Element	Mass Fraction/%
C	6.91
Cr	2.09
Ni	3.52
Fe	Bal.

**Table 4:** Quantitative and qualitative phase fraction of martensite/bainite via EBSD for TAC and TWC.

Tempering Cooling Method	Martensite	Bainite
Tempering air cooling	60%	40%
Tempering water cooling	67%	33%

## Data Availability

Data are contained within the article.
